# Metataxonomic analysis of tissue-associated microbiota in grooved carpet-shell (*Ruditapes decussatus*) and Manila (*Ruditapes philippinarum*) clams

**DOI:** 10.1007/s10123-021-00214-9

**Published:** 2021-10-04

**Authors:** Diego Gerpe, Aide Lasa, Alberto Lema, Jesús L. Romalde

**Affiliations:** Departamento de Microbiología Y Parasitología, CRETUS & CIBUS-Facultad de Biología, Universidade de Santiago de Compostela, Campus Vida s/n, 15782 Santiago de Compostela, Spain

**Keywords:** Clams, Microbiota, Tissue associated, Population structure, Seasonality

## Abstract

Culture-dependent techniques only permit the study of a low percentage of the microbiota diversity in the environment. The introduction of next generation sequencing (NGS) technologies shed light into this hidden microbial world, providing a better knowledge on the general microbiota and, specifically, on the microbial populations of clams. Tissue-associated microbiota of *Ruditapes decussatus* and *Ruditapes philippinarum* (mantle, gills, gonad and hepatopancreas) was analysed in two different locations of Galicia (northwest of Spain) during Spring (April) and Autumn (October), employing a metataxonomic approach. High bacterial diversity and richness were found in all samples where a total of 22,044 OTUs were obtained. In most samples, phylum *Proteobacteria* was most frequently retrieved, although other phyla as *Actinobacteria*, *Bacteroidetes*, *Tenericutes*, *Firmicutes* or *Chlamydiae* also appeared at high relative abundances in the samples. At genus level, great variation was found across tissues and sampling periods. A Nonmetric Multidimensional Scaling (NMDS) and a hierarchical clustering analysis allowed to further analyse the factors responsible for the differences among groups of samples in the different sites. Results showed sample ordination based on tissue origin and sampling periods, pointing out that the microbiota was influenced by these factors. Indeed, predominance of certain genera was observed, such as *Endozoicomonas* or *Methylobacterium* in gills and gonads, respectively, suggesting that selection of specific bacterial taxa is likely to occur. So far, this study provided a general picture of the tissue associated microbial population structure in *R. decussatus* and *R. philippinarum* clams, which, ultimately, allowed the identification of specific tissue-related taxa.

## Introduction

The culture of shellfish, specially clams and mussels, represents a key economic activity in the aquaculture of Galicia, a region in the North West of Spain. The overexploitation of natural beds led to the introduction of seeds and adult specimens from other countries, increasing the risk of introducing new bacterial pathogens that might disrupt the natural microbiota of the coastal environment, as well as the microbial communities associated to marine organisms, such as molluscs (Bower et al. 1994).

Bivalve molluscs are powerful filter feeding organisms, allowed to filter large volumes of water, while concentrating different microorganisms. Bivalves, such as clams, are capable of retaining an important bacterial fraction in their organism, namely associated microbiota, while expelling to the environment the so-called microbiota in transition. Symbiotic associations, as a result of complex interactions, between bacteria and molluscs are well documented after selection upon the great marine microbial diversity (Mandel and Dunn 2016; Yu et al. 2019). Indeed, environmental microbiome play an important role in the formation and structure of the host-microbe complex (Singh et al. 2020). The associated microbiota plays different roles in bivalve molluscs, some of which may be beneficial for the host while others may have harmful effects (McHenery and Birkbeck 1985; Prieur et al. 1990; Seguineau et al. 1996; Romalde et al. 2013, 2014).

Some studies focused on microbial communities in molluscs have analysed the variation of the bacterial population structure taking into account spatial (Colwell and Liston 1961; Colwell and Sparks 1967; Lovelace et al. 1968) and temporal variables (Pujalte et al. 1999), different phases of growth of the bivalve or the different composition of the microbiota in their organs (Rajagolapan and Sivalingan 1978). Most studies regarding microbiota of bivalve molluscs were based on the culturable fraction of bacteria and on the detection of pathogenic species (Paillard et al. 2004; Romanenko et al. 2008; Balboa et al. 2016). Recently, Next-Generation Sequencing (NGS) technologies have been introduced in the studies of the microbiota associated to different bivalve species, showing the high bacterial diversity present in the studied mollusc species. Some of these studies have focused on the analysis of whole-body homogenates (Trabal Fernández et al. 2014), single tissues (Lokmer and Wegner 2015; Roterman et al. 2015; Lasa et al. 2016) or compared the microbiota composition between different tissues (Lokmer et al. 2016; Vezzulli et al. 2017).

A study focused on oyster microbiota (*Crassostrea corteziensis*, *Crassostrea gigas* and *Crassostrea sikamea*) revealed a complex community consisting of 13 phyla and 243 genera associated with these molluscs in different life stages, where *Proteobacteria* was the predominant phylum in all stages. In postlarvae, the most relative abundant genera were *Neptunibacter*, *Marinicella*, *Rhodovulum* and *Oceanicola*, while in adults the dominant genera were *Burkholderia* and *Escherichia*-*Shigella* (Trabal Fernández et al. 2014). Other study based on the microbiota associated to *Pecten maximus* gonads (Lasa et al. 2016) had similar results and described 13 phyla and 110 genera, including *Delftia*, *Acinetobacte*r, *Hydrotalea*, *Aquabacterium*, *Bacillus*, *Sediminibacterium*, *Sphingomonas* and *Pseudomonas*, as the most relative abundant taxa. More recently, a study conducted on the haemolymph and digestive gland microbiota of *Mytilus galloprovincialis* and *C. gigas* also revealed the predominance of phylum *Proteobacteria*, being *Vibrio* and *Pseudoalteromonas* the most retrieved genera in both bivalve species (Vezzulli et al. 2017).

Grooved carpet shell clam (*Ruditapes decussatus*) and Manila clam (*Ruditapes philippinarum*) are the more important reared clam species in Spain. So far, studies analysing the associated microbiota of these two species were focused in the culturable bacteria where the predominant genera were *Vibrio* and *Pseudoalteromonas* (Romalde et al. 2013; Leite et al*.* 2017).

In the present study, a metataxonomic analysis was performed on two different clam species, *R. decussatus* and *R. philippinarum*, gathered in Spring and Autumn in two different sites in the Galician coast (Redondela and Carril) to unravel the tissue-associated microbial population structure.

## Materials and methods

### Sample collection

**Fig. 1:**
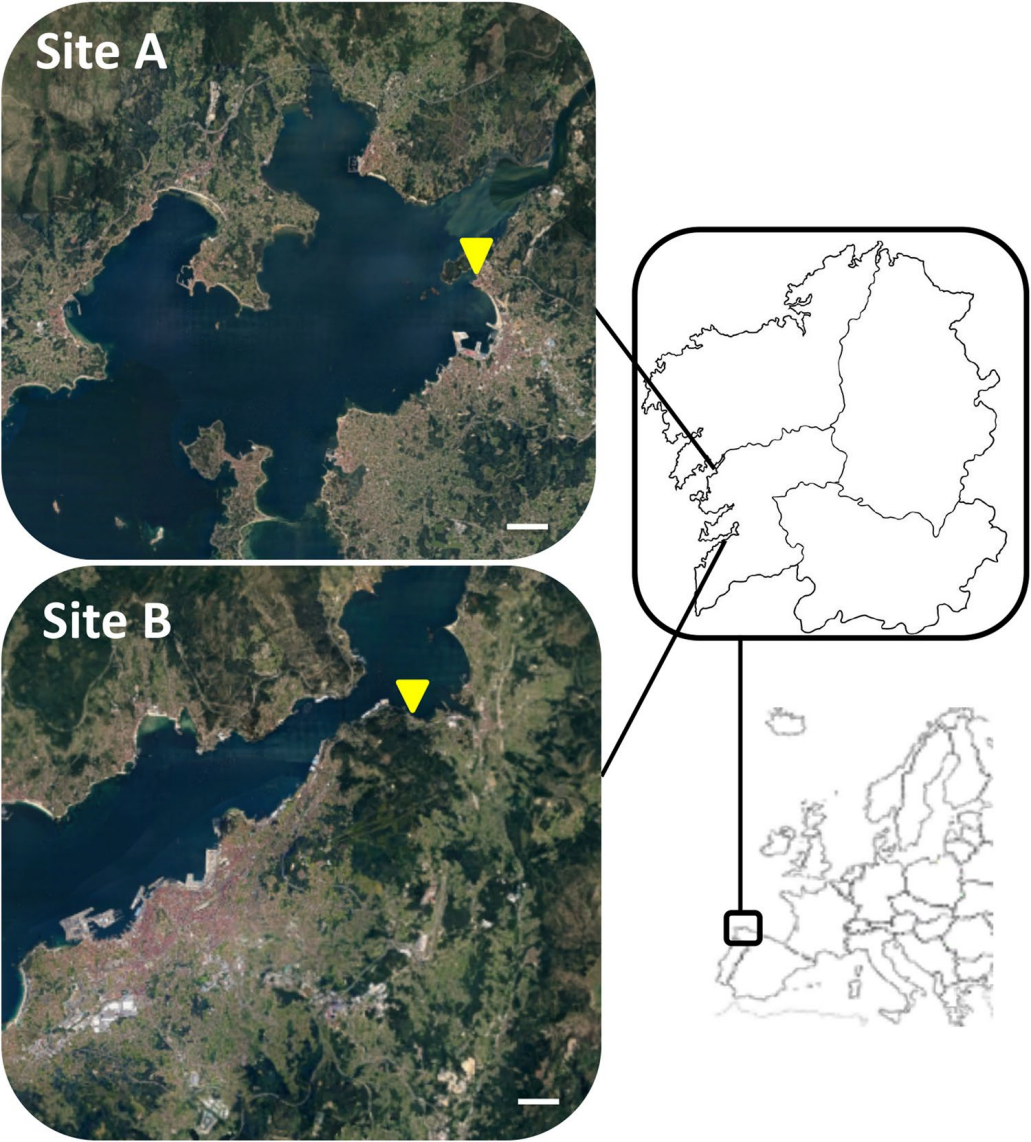
Geographical map of Galicia (NW Spain) showing the two selected sampling sites. Source of colour pictures: Google Earth (data accession July 12^th^, 2021)

Two different species of reared clams (*R. decussatus* and *R. philippinarum*) were selected to analyse the associated microbiota to the different tissues (gonad, hepatopancreas, gills and mantle). The specimens (*n* = 25 of each species) were collected in site A (42°36′50.4″ N 8°46′39.1″ W) and site B (42°17′40.4″ N 8°36′57.2″ W) and in two different periods, Spring (April) and Autumn (October) (Fig. 1). In Spring, the registered water temperature was 13 °C in site A and 13.7 °C in site B, while in Autumn temperature of the water was 15.2 °C in site A and 16.5 °C in site B. Immediately after collection, clam samples were transported to the laboratory in a refrigerator at 4 °C, approximately during 3 h.

### Tissue homogenization

The clams were aseptically opened for the extraction of the tissues (gonad, hepatopancreas, gills and mantle) using a sterile scalpel. Then, each tissue from the 25 specimens was pooled and homogenized in PBS. Briefly, bacterial cells of each sample were separated from eukaryotic cells and concentrated using a gradient centrifugation. This gradient was performed using OptiPrep™ (Sigma), a medium of gradient density composed of 60% of iodinaxol. OptiPrep™ was diluted with 0.25 M sucrose, 6 mM EDTA, 60 mM Tris–HCl, pH 7.4 to produce 14%, 25% and 55% (w/v) iodixanol. Four millilitres of each concentration of iodixanol was layered, and 10 ml of each sample was layered on top. A first centrifugation was made at 3.800 × g at 4–10 °C for 60 min. Then, the upper layer was collected in other tube and centrifuged again at 3000 × g at 4 °C for 60 min. The pellet was washed with 2 ml of PBS by centrifugation and centrifuged during 30 min at 13,000 × g. Finally, the pellet was resuspended in 1 ml of PBS and stored at − 20 °C until DNA extraction.

### DNA extraction and PCR amplification

DNA was extracted using the MasterPure Complete DNA and RNA Purification kit (Epicentre Biotechnologies) following the manufacturer’s instructions. DNA concentration and quality were determined by agarose gel electrophoresis (1% wt/vol agarose in Trisacetate-EDTA buffer) and using NanoDrop ND-1000 spectrophotometer (Thermo Scientific). Extracted DNA was stored at − 20 °C until use for PCR amplification.

Genomic DNA from each sample was used for the amplification of 16S rRNA gene using primers targeting the hypervariable regions V3/V4 (Lee et al. 2012): 338F (5′- TCGTCGGCAGCGTCAGATGTGTATAAGAGACAGACTCCTACGGGAGGCAGCA-3′) and 806R (5′GTCTCGTGGGCTCGGAGATGTGTATAAGAGACAGGGACTACHVGGGTWTCTAAT-3′). Sterilized MilliQ water was included as a negative control. The 16S rRNA amplicons were verified by gel electrophoresis on a 2% agarose gel using GreenSafe DirectLoad (NZytech) for the staining. DNA concentration was determined using a NanoDrop ND-1000 spectrophotometer (Thermo Scientific).

### 16S rRNA amplicon sequencing and data analysis

16S amplicons were sequenced at Sistemas Genómicos (Valencia, Spain) using Illumina MiSeq platform, generating paired-end 2 × 250 bp reads.

Illumina reads were analysed for quality control using FastQC software (Brabaham Bioinformatics). Quality trimming of reads was performed based on quality scores (Q < 30) and length trimming (200 base pairs-bp), using Trimmomatic 0.32 (Bolger et al. 2014) program, as well as chimera detection and removal. The filtered paired-end reads were then merged using the command fastq-join (Quast et al. 2013) and clustered at 97% level of similarity into OTUs. Ribosomal RNA gene reads were classified against the non-redundant version of the SILVA SSU reference taxonomy (release 123; http://www.arb-silva.de). For bacterial diversity estimation in the samples, the number of operational taxonomic units (OTUs) at 97% sequence identity was determined, and rarefaction analyses were carried out. Briefly, the reads were aligned against the 16S rRNA sequences of the SILVA database followed by a quality filtering including length, ambiguity and homopolymer checks. A de-replication step was performed to collapse identical reads into one single sequence, and OTUs were clustered at 3% divergence threshold. The mitochondria, chloroplasts and unassigned reads were deleted for the taxonomic analysis.

### Statistical analysis

Nonmetric Multidimensional Scaling (NMDS), hierarchical clustering and analysis of similarities (ANOSIM) were performed from the dissimilarity matrix using vegan package of R (Clarke 1993, Oksanen et al. 2017). Heatmap was also performed using pheatmap package (v. 1.0.12, Kolde 2015).

## Results

**Fig. 2:**
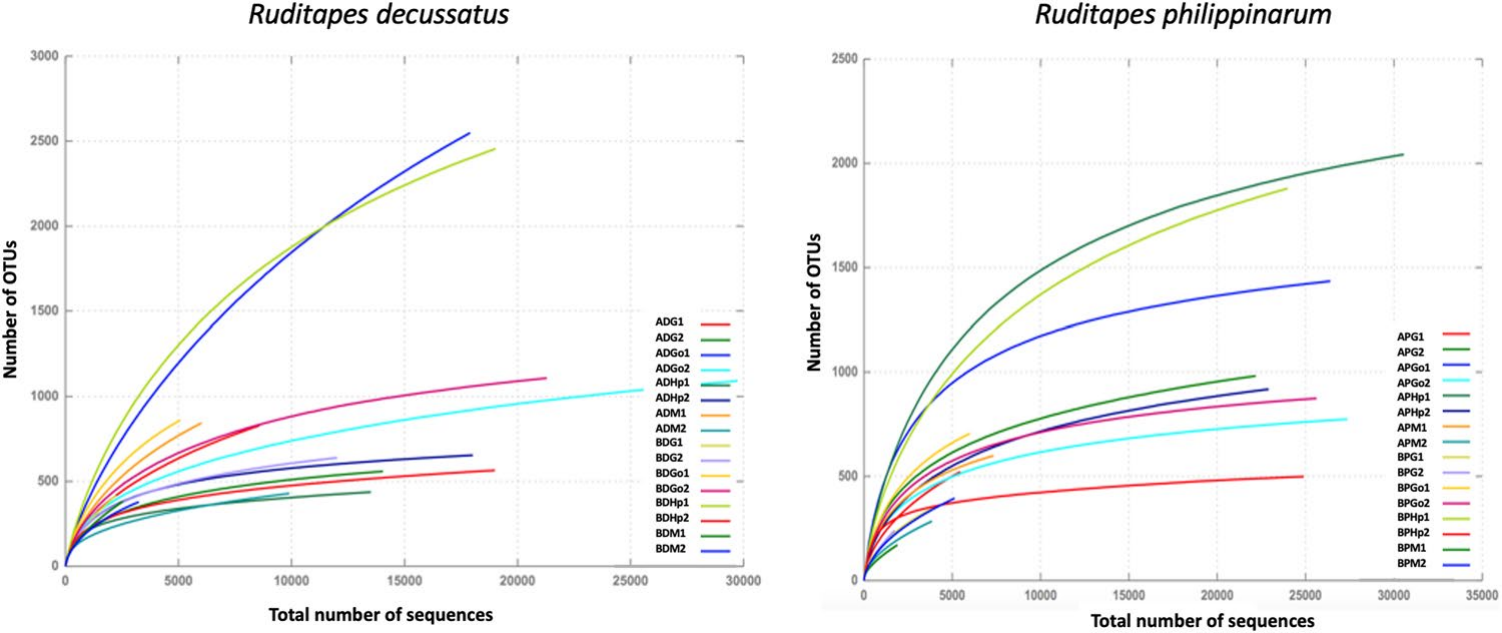
Rarefaction analysis of samples of *R. decussatus* and *R. philippinarum*, showing the number of OTUs (at 97% 16S rRNA gene sequence identify) as a function of the number of sequences analysed. A site A, B site B, D *R. decussatus*, P *R. philippinarum*, M mantle, G gills, Go gonads, Hp hepatopancreas, 1 April, 2 October

After filtering raw sequences obtained from the V3/V4 region of 16S rRNA, a total of 441,672 reads were obtained from samples of *R. decussatus and R. philippinarum* with an average length from 453 to 460 pb. A total of 22,044 OTUs were obtained, of which more than 93% of clustered sequences could be taxonomically assigned (Table 1), except for samples ADHp1, APG1, BPG1 and BPG2. Rarefaction analysis (at 97% sequence identity level) (Fig. 2) of *R. decussatus* and *R. philippinarum* samples showed that most samples reached saturation or asymptotic phase.

**Table 1:** Summary of the characteristics of all samples of *R. decussatus* and *R. philippinarum*, sequences analysed and diversity/richness indexes

Sample	Sampling period	Site	Number of sequences	Avg. length	OTUs	% classified	Number of sequences observed once	Number of sequences observed twice	Chao-1
ADM 1	April	A	6024	463	844	97.44	409	159	1365.48
ADG 1	April	A	8583	465	829	99.11	393	143	1363.92
ADGo 1	April	A	17,903	454	2549	98.91	1324	504	4283.31
ADHp 1	April	A	13,526	454	437	99.65	114	38	602.15
ADM 2	October	A	9929	466	429	99.68	164	59	651.77
ADG 2	October	A	14,102	457	559	99.26	139	83	673.18
ADGo 2	October	A	29,734	460	1092	99.90	356	169	1463.71
ADHp 2	October	A	18,020	464	654	72.79	127	70	766.69
BDM 1	April	B	2532	462	381	94.08	227	51	874.29
BDG 1	April	B	2231	458	417	94.04	251	60	931.34
BDGo 1	April	B	5105	458	861	97.90	378	190	1234.05
BDHp 1	April	B	19,036	460	2455	97.53	902	469	3319.58
BDM 2	October	B	3251	459	379	99.14	178	67	610.66
BDG 2	October	B	12,018	457	639	99.60	179	91	812.16
BDGo 2	October	B	21,290	451	1108	99.50	284	159	1359.16
BDHp 2	October	B	18,994	451	565	99.58	151	53	774.72
APM 1	April	A	7340	462	599	98.83	178	93	766.59
APG 1	April	A	5462	462	522	72.28	245	103	809.40
APGo 1	April	A	26,402	457	1434	93.41	252	133	1670.01
APHp1	April	A	30,581	461	2042	99.36	441	284	2382.42
APM 2	October	A	3887	466	285	98.56	153	35	608.00
APG 2	October	A	22,202	462	982	99.63	272	108	1320.13
APGo 2	October	A	27,372	454	774	99.88	150	87	900.99
APHp 2	October	A	22,915	456	918	98.66	241	125	1147.52
BPM 1	April	B	1899	463	171	88.94	103	19	433.65
BPG 1	April	B	3912	465	350	68.71	178	49	665.06
BPGo 1	April	B	6003	456	706	98.23	246	129	937.81
BPHp 1	April	B	24,009	460	1879	97.62	578	307	2420.41
BPM 2	October	B	5153	462	395	88.03	206	57	759.05
BPG 2	October	B	1738	464	240	47.01	147	36	530.03
BPGo 2	October	B	25,621	454	874	94.02	155	106	985.54
BpHp 2	October	B	24,898	454	499	96.61	94	39	608.28

*A* site A, *B* site B, *D R. decussatus*, *P R. philippinarum*, *M* mantle, *G* gills, *Go* gonads, *Hp* hepatopancreas, *1* April, *2* October

**Fig. 3:**
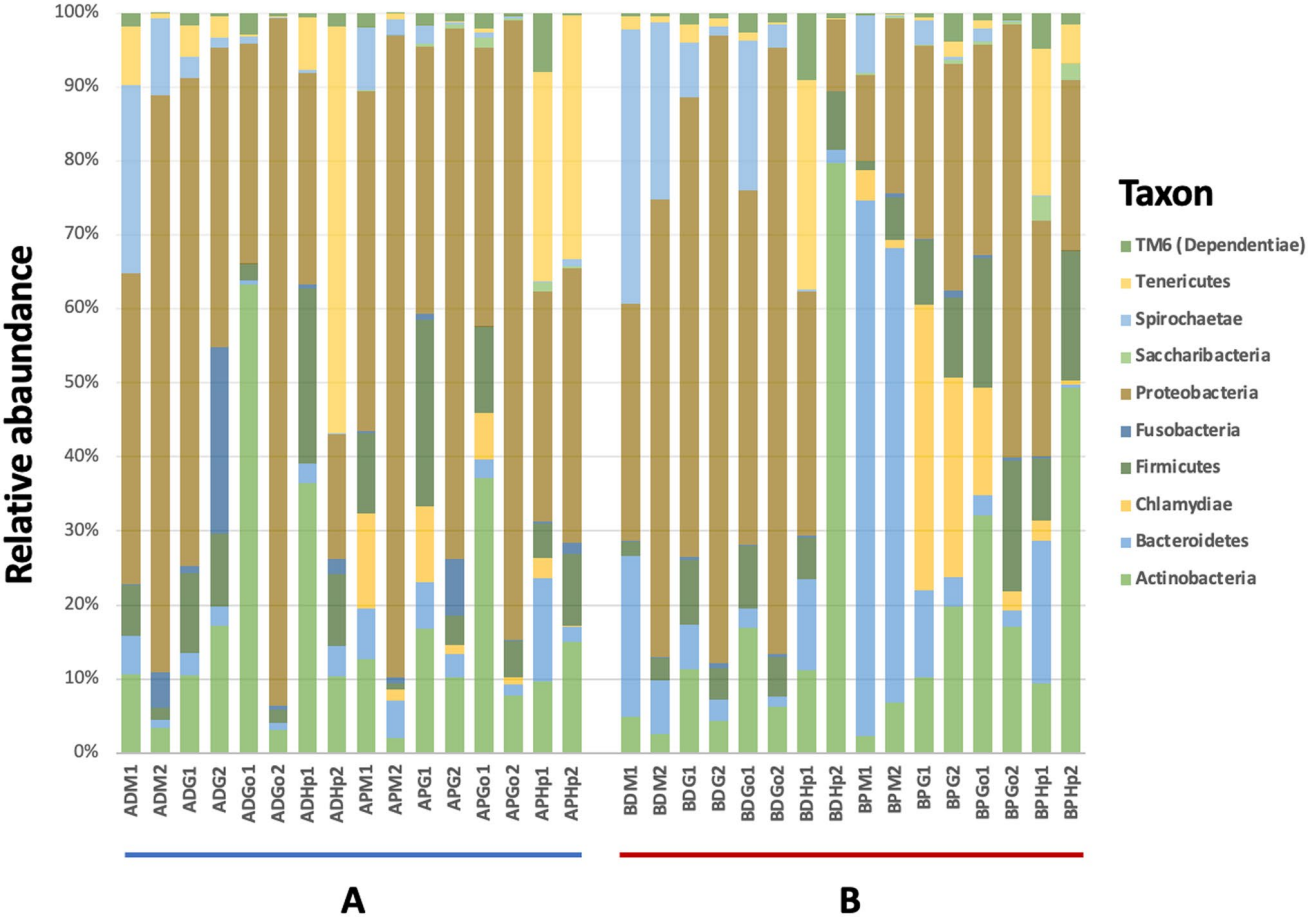
Relative abundances of bacterial phyla associated to *R. decussatus* and *R. philippinarum* organs indicating sampling sites A and B. The graphic shows the percentages (> 1%) of the 16S rRNA reads assigned to different bacteria taxa. A site A, B site B, D *R. decussatus*, P *R. philippinarum*, M mantle, G gills, Go gonads, Hp hepatopancreas, 1 April, 2 October

Taxonomic assignment of the sequences using the SILVAngs database identified more than 30 different phyla within the two clam species microbial communities. Despite the high number of phyla detected, more than 90% of the observed diversity can be explained taking into account only 14 phyla that dominated the total bacterial population (Fig. 3). Among them, *Proteobacteria*, *Firmicutes*, *Actinobacteria*, *Bacteroidetes* or *Spirochaetae* account for the major fraction on every sample, but displaying compositional variations depending on the tissue, sampling period or clam species. For instance, samples gathered in site A harboured, predominantly, bacteria belonging to *Proteobacteria* (ranging from 15.6 to 91.6%) in both clam species. However, for samples ADGo1, ADHp1 and ADHp2, the major fraction of the bacterial population could be explained by *Actinobacteria* (58.4% and 35.4%) and *Tenericutes* (50.8%) respectively (Fig. 3). On the other hand, samples taken in site B displayed different bacterial communities depending on the clam species and tissue. Grooved carpet-shell clams showed similar bacterial phyla composition to that in site A, composed by *Proteobacteria* mainly (ranging from 29.8 to 82.6%), although the BDM1 sample showed a more diverse bacterial population formed by *Spirochaetae* (36.2%), *Proteobacteria* (31.2%) and *Bacteroidetes* (21.2%). Manila clam samples from site B showed a different microbial population pattern, and *Proteobacteria* was the predominant phylum only in BPG2 (30.2%), BPGo2 (57.1%) and BPHp1 (29.4%) samples. Conversely, mantle samples were enriched in phylum *Bacteroidetes* (71.9% and 60.8%), and the BPG1 sample was dominated by phylum *Chlamydiae* (37.8%). Besides, BPGo1 and BPHp2 samples showed the predominance of *Actinobacteria*, 31.2% and 48.9% respectively (Fig. 3).

### Tissue specificity and seasonal variation

**Fig. 4:**
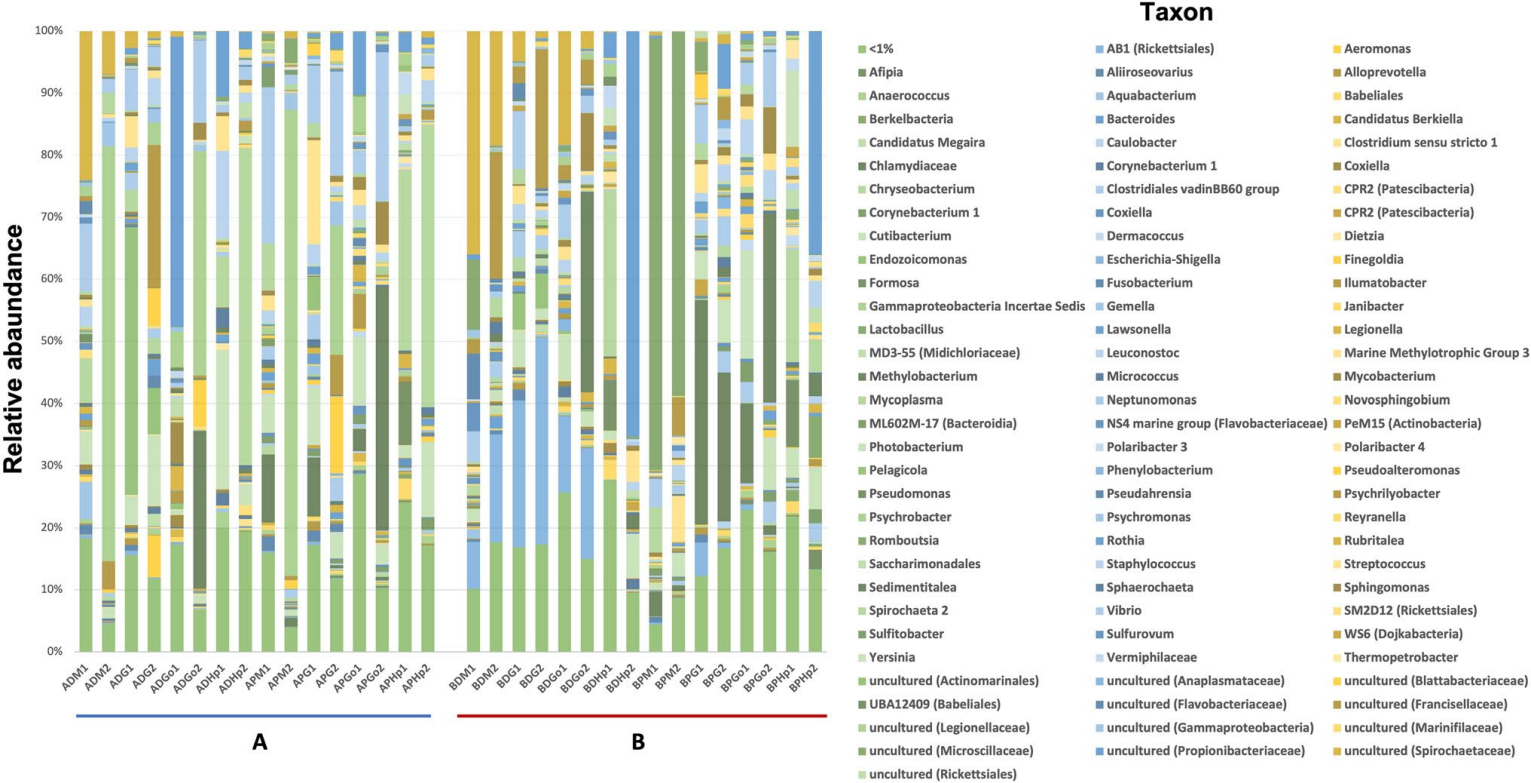
Relative abundances of bacterial genera associated to *R. decussatus* and *R. philippinarum* organs indicating sampling sites A and B. The graphic shows the percentages (> 1%) of the 16S rRNA reads assigned to different bacterial taxa. A site A, B site B, D *R. decussatus*, P *R. philippinarum*, M mantle, G gills, Go gonads, Hp hepatopancreas, 1 April, 2 October

16S rRNA amplicon analysis at the genus level showed distinct relative abundances across tissues in both clam species. Relative abundance differences observed in specific bacterial genera may indicate that selection of specific bacterial groups upon the great microbial diversity in the marine environment is likely to occur. For instance, *Endozoicomonas* genus appeared in gill samples of grooved carpet shell clams in both sites at high relative abundances, especially in sample ADG1 (43%), while this genus was not detected or at very low concentrations (< 1%) in other tissues and *R. philippinarum* clams. Similarly, gonad samples from the two different clam species at both sites were enriched in *Methylobacterium* genus, representing 25.3%, 39.1%, 32.1% or 30.5% in ADGo2, APGo2, BDGo2 and BPGo2 samples, respectively (Fig. 4).

Uncultured *Microscillaceae* was the predominant taxon in mantle samples of Manila clams from site B in both periods, accounting for more than 59% of relative abundance. Conversely, grooved carpet shell clam samples from site B showed high concentrations of AB1 *Rickettsiales* (7.5 to 33.3%) in all studied organs and sampling periods, except in hepatopancreas in which, this taxon, was not detected.

Uncultured *Propionibacteriaceae* were more related to gonad and hepatopancreas samples from site A during Spring (46.7 to 3.1%) in both clam species, while in site B this group was predominately related to Hepatopancreas tissue during the second sampling period (65.2 to 35.4%). Another genus detected specially in hepatopancreas samples is *Mycoplasma*. In site A, an increase was observed from Spring to October, from 8.1 to 51% (*R. decussatus*) and from 28.9 to 45.5% (*R. phillipinarum*). On the other hand, *Mycoplasma* was more abundant in hepatopancreas samples during Spring in site B.

Sampling period (Spring and Autumn) also influenced bacterial community structures associated to the different tissues. It is well known that water temperature shapes marine microbiota, and, as a consequence of their filter-feeding habit, clam microbiota is affected, too. This appears evident for the genus *Psychrobacter* in samples of mantle, gills and gonads from site A, from both *R. decussatus* and *R. philippinarum* species, which relative abundances are increased during the second sampling period (Autumn). Similar trend is observed in *Methylobacterium* genus in which we observed an increase during Autumn period in gonad samples compared to Spring period. Conversely, *Chlamydiaceae* bacterial group resulted more abundant in Spring samples (APM1, APG1, APGo1, BPM1, BPG1 or BPGo1) than in Autumn samples.

**Fig. 5:**
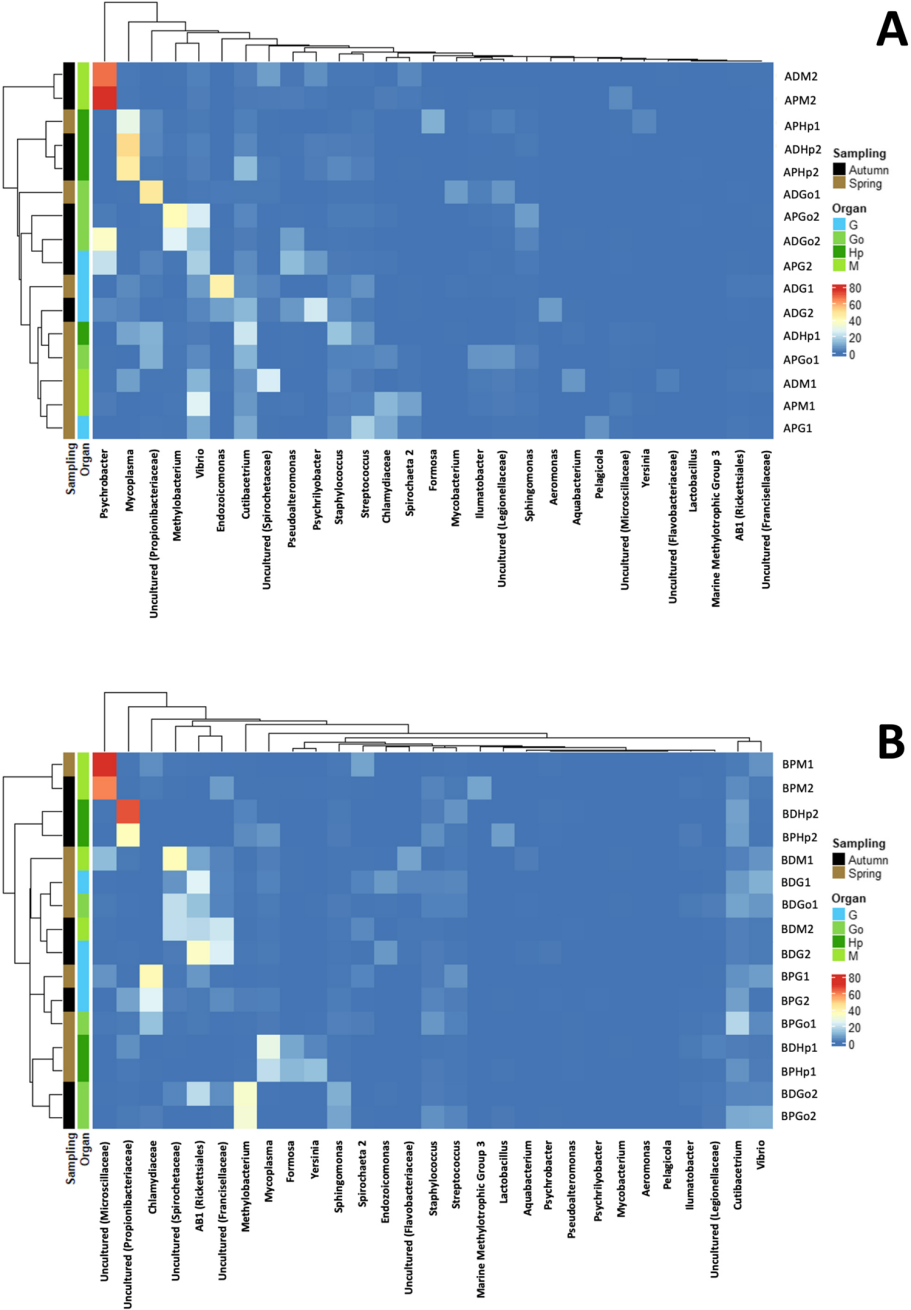
Hierarchical clustering dendrogram of microbiota associated to clam tissues. The heatmap depicts the relative abundance of each genus in each sample applied on ANOSIM distance matrix. **A** Dendrogram of site A. **B** Dendogram of site B. The colour scale, tissue and sampling period for the heatmap is displayed in the right side of the figure. A site A, B site B, D *R. decussatus*, P *R. philippinarum*, M mantle, G gills, Go gonads, Hp hepatopancreas, 1 April, 2 October

These findings were confirmed when a hierarchical clustering of the most abundant genera (> 5% relative abundance), separately in both sites, was performed. The resulting heatmap showed that genera correlated well with their association to tissues or the sampling period, as depicted in Fig. 5. In general, samples in site A belonging to the same organ clustered together except for samples ADHp1, APGo1, ADM1, APM1, APG1, which appear to be more affected by environmental factors. This is less evident in site B, in which the sampling period seemed to be the responsible of the sample clustering. These results suggest that both variables are involved in defining the associated microbial communities in both clam species.

**Fig. 6:**
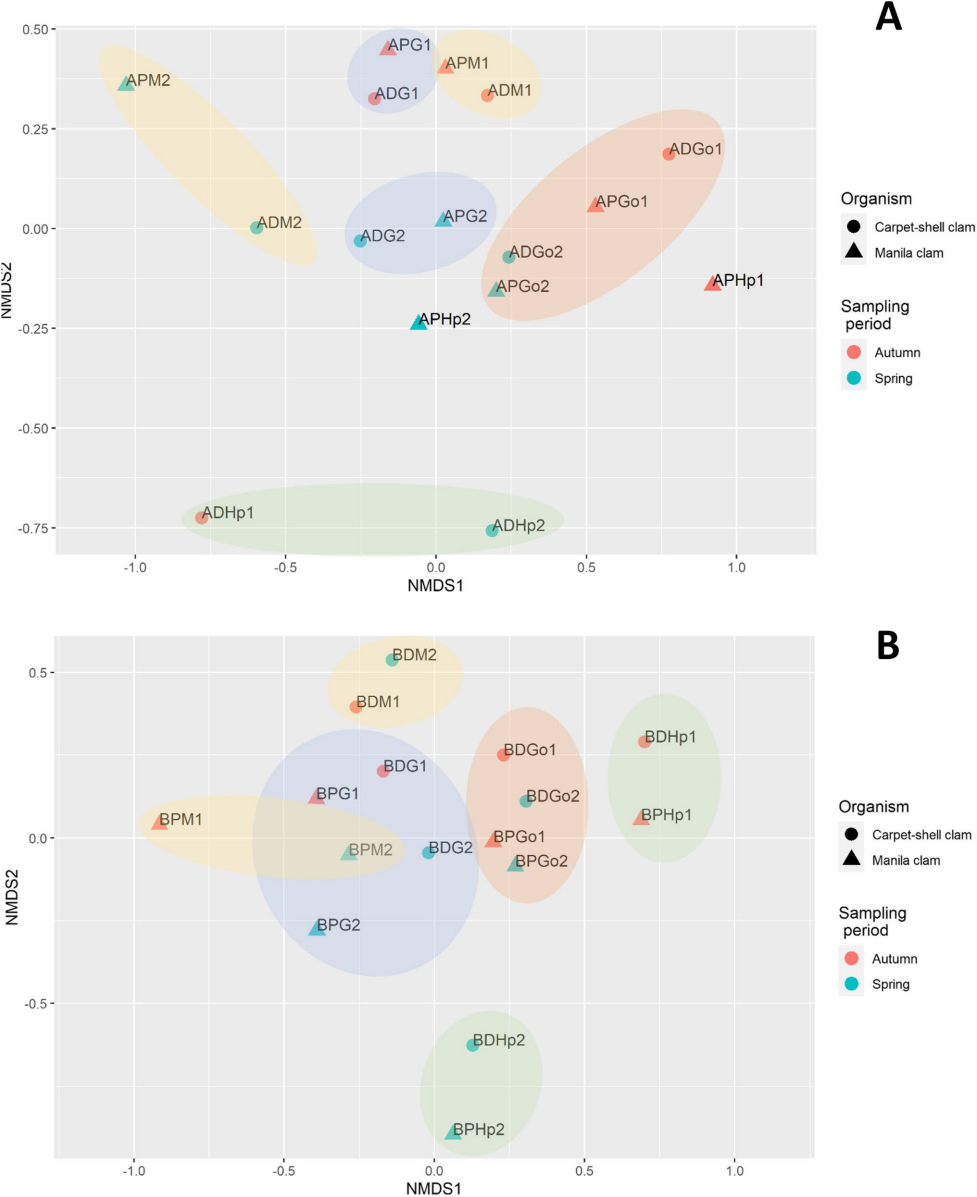
2D representation of Nonmetric Multidimensional Scaling (NMDS) plots applied on ANOSIM distance matrix. Ellipses indicate group samples by tissue. **A** NDMS plot of site A. **B** NMDS plot of site B. A site A, B site B, D *R. decussatus*, P *R. philippinarum*, M mantle, G gills, Go gonads, Hp hepatopancreas, 1 April, 2 October

Microbial composition differences have been reflected, as well, when a Nonmetric Multidimensional Scaling (NMDS), applied on ANOSIM distance matrix, analysis was performed. This analysis led to further investigate the factors responsible for differences among group of samples in the different sites. In site A, samples appeared to be ordinated based on sampling period and tissue origin rather than on clam species (Fig. 6A), except hepatopancreas samples that were ordinated based on the clam species, although every sample was separated further apart one to each other. In contrast, site B samples were ordinated according to clam species and by organs, as well, except for hepatopancreas samples which were more similar between sampling periods (Fig. 6B). Gill and gonad samples clustered together, while hepatopancreas and mantle samples were clearly separated based on clam species and sampling period, respectively.

### Influence of habitat on clam microbiota

When analysing the data obtained on the basis of the origin of sampling (A or B), it becomes clear that the environment of these two locations is playing an important role shaping the associated microbiota of *R. decussatus* and *R. philippinarum* clams. Differences at the genus level are evident when comparing the microbiota of the same clam species from different locations. For instance, in site B, grooved carpet shell clam displayed a stable relative abundance of AB1 *Rickettsiales* group throughout the different organs (except in hepatopancreas), while in site A this genus appears at low concentrations (< 1%) (Fig. 4). Similarly, genus *Psychrobacter* is mainly associated to *R. decussatus* and *R. philippinarum* clams in site A (Fig. 5) at high relative abundances, ranging from 20.6 to 75.5%, (ADM2, APM2, ADGo2 or APG2), but in site B its presence is clearly reduced (lower than 1%). Despite these differences, bacterial groups such as *Methylobacterium* or *Endozoicomonas*, which appear to be positively selected in gonad and gill samples respectively, seemed not to be affected by the different environments. Mantle and hepatopancreas bacterial populations displayed considerable differences between locations. Mantle and hepatopancreas in site B were enriched in uncultured *Microscillaceae* and uncultured *Propionibacteriaceae* taxa, while *Psychrobacter* and *Mycoplasma* were dominant in those tissues in site A location (Fig. 5A). Vibrios were also identified in all samples; however, their relative abundance was higher in site A, ranging from 25.9 to 0.8%, while in site B their presence is clearly reduced, ranging from 9.3 to 0.4%.

## Discussion

In the present study, we investigated the organisation of the tissue-associated microbiota of Grooved carpet shell clam, *R. decussatus,* and Manila clam, *R. philippinarum*, in two different locations (A and B) and in two periods, Spring and Autumn. A considerable heterogeneity among individuals was demonstrated for other bivalves, such as pearl oyster (King et al. 2021). In our work, in order to get a solid overview of the bacterial communities associated with clam populations that could be useful for the determination of their sanitary status, pooled samples were employed to avoid possible microbial composition changes due to individual specimen variations. We found that each analysed tissue was composed, in terms of taxonomical composition and structure, of distinct bacterial communities. Besides, microbiota composition fluctuated between sampling periods.

Host-associated microbiota consist of more or less complex communities of microorganisms, some of which are more adapted to their host, other generalists or transient, representing a wide range of potential contributions (Shapira 2017). They play a key role in host homeostasis and health, by promoting development (McFall-Ngai 2002), providing protection against pathogens (Offret et al. 2018) or improving adaptation to environmental changes (Torda et al. 2017). It is well known that bivalves harbour their own microbiota (as for other organisms), whose characteristics and functions are still poorly understood, but cannot be ignored (Desriac et al. 2014; Offret et al. 2019a, b).

Our study contributes to widen the knowledge about the clam-associated microbiota and, specially, the microbial structure population at the tissue level. We observed that the associated microbiota at tissue level consisted of a great bacterial diversity (Fig. 3) which, in origin, belonged to the marine environment, although host selection ultimately shaped the microbiota structure (Fig. 6A, B). Meisterhans et al. (2016) investigated Manila clam-associated microbiota at organ scale (gills, gut and a pool of remaining tissues), and they found that microbiota structure differed among organs indicating a selection of Manila clam microbiota at organ scale, which agrees with our results.

Despite variability across conditions, certain stability and specificity were observed in the different studied tissues, as we observed with *Endozoicomonas*, *Methylobacterium*, *Mycoplasma*, *Psychrobacter* or uncultured *Propionibacteriaceae*. The ecological role or potential symbiotic association of most of the bacterial groups identified and the host remains unclear; however, recent studies on marine symbionts have shed light into this question.

*Endozoicomonas* genus, mostly associated to gill samples in *R. decussatus* clams, has been associated as symbionts with a large diversity of marine organisms including cnidarians, poriferans, other molluscs, annelids, tunicates and fish (Neave et al. 2016). Despite the abundance of *Endozoicomonas* symbionts, only seven complete genomes are publically available, thus, limiting the understanding of their functional capacity. A comparative genomic study on *Endozoicomonas* (Neave et al. 2017) provided a deep functional insight into this genus. Genomic content showed an enrichment of genes associated with carbon sugar transport and utilization and protein secretion, potentially indicating that *Endozoicomonas* contribute to the cycling of carbohydrates and the provision of proteins to their respective hosts. Besides, *Endozoicomonas* genomes were enriched in transposition and DNA recombination systems, which may help the species to rapidly adapt to a new host or to opportunistically transition between symbiotic lifestyles (mutualistic, commensalistic or parasitic). Our results demonstrate that *R. decussatus* gills may represent an optimal environment for *Endozoicomonas* bacteria to survive in association with these clams by establishing complex interactions and providing a diversity of beneficial effects to the host.

*Methylobacterium* are facultative methylotrophic phytosymbionts that can utilize formaldehyde, methanol, methylamine or other methyl compounds, as sole carbon and energy source. Species of this genus have been often found associated to plants (they are able to produce phytohormones), mussels, soil or even extreme environments such as gamma ray-irradiated soil and a tungsten mine (Kim et al. 2019; Feng et al. 2020; Jia et al. 2020; Jiang et al. 2020); however, they have never been found before associated to clams. Their strong association to gonad samples suggests their potential implication in the protection of this particular organ or their ability to provide beneficial substances; however, these questions have not been addressed yet.

Recent studies have demonstrated that the bivalve microbiota is highly diverse and easily influenced by environmental factors, such as water temperature, pH, salinity, dissolved oxygen, nutrients and infections (e.g. Green and Barnes 2010; Lokmer and Wegner 2015). Changes in the water temperature between sampling periods, as indicated above, might explain the variation in the relative abundances of the identified bacteria, that, indeed, is reflected in the hierarchical clustering (Fig. 5A,B) and the NMDS analysis (Fig. 6A,B). However, it remains unclear whether the compositional variation is due to the direct effect of water temperature in water microbial communities or as a result of the change on the physiology of *R. decussatus* and *R. philippinarum* clams, or both.

Clams from both sites harboured different bacterial communities associated to their organs. This result was expected since both habitats are geographically isolated one to each other, and, thus, different sediment conditions and trophic resources from which clams feed might be present. These aspects have a direct effect on both clam physiology and the bacterial suspension in the environment.

## Conclusions

In light of our results, the microbial community structure of *R. decussatus* and *R. philippinarum* clams appears to be tissue dependent. Genera are shared across samples; however, differences in their relative abundances are significative, indicating that host selection of specific bacteria may occur. Seasonal changes and habitat influence are also observed in the bacterial composition, and, thus, these variables must be taken into account when analysing the health status of the clams.

## Data Availability

Sequence files for all samples used in this study have been deposited at NCBI SRA with accession: PRJNA428215.
